# Bioefficacy and durability of Olyset^®^ Plus, a permethrin and piperonyl butoxide-treated insecticidal net in a 3-year long trial in Kenya

**DOI:** 10.1186/s40249-021-00916-2

**Published:** 2021-12-20

**Authors:** Paul M. Gichuki, Luna Kamau, Kiambo Njagi, Solomon Karoki, Njoroge Muigai, Damaris Matoke-Muhia, Nabie Bayoh, Evan Mathenge, Rajpal S. Yadav

**Affiliations:** 1 Eastern & Southern Africa Centre of International Parasite Control, Kenya Medical Research Institute, Nairobi, Kenya; 2 School of Health Sciences, Meru University of Science and Technology, Meru, Kenya; 3 Centre for Biotechnology Research and Development, Kenya Medical Research Institute, Nairobi, Kenya; 4 Division of National Malaria Programme, Ministry of Health, Nairobi, Kenya; 5 Department of Health, Kirinyaga County, Kirinyaga, Kenya; 6 Centre for Global Health Research, Kenya Medical Research Institute, Kisumu, Kenya; 7 Centers for Disease Control and Prevention, Kisumu, Kenya; 8 Department of Control of Neglected Tropical Diseases, World Health Organization, Geneva, Switzerland

**Keywords:** *Anopheles gambiae*, Bioefficacy, Durability, Kenya, Long-lasting insecticidal net, Olyset^®^ Net, Olyset^®^ Plus, Permethrin, Piperonyl butoxide

## Abstract

**Background:**

Long-lasting insecticide nets (LLINs) are a core malaria intervention. LLINs should retain efficacy against mosquito vectors for a minimum of three years. Efficacy and durability of Olyset^®^ Plus, a permethrin and piperonyl butoxide (PBO) treated LLIN, was evaluated versus permethrin treated Olyset^®^ Net. In the absence of WHO guidelines of how to evaluate PBO nets, and considering the manufacturer’s product claim, Olyset^®^ Plus was evaluated as a pyrethroid LLIN.

**Methods:**

This was a household randomized controlled trial in a malaria endemic rice cultivation zone of Kirinyaga County, Kenya between 2014 and 2017. Cone bioassays and tunnel tests were done against *Anopheles gambiae* Kisumu. The chemical content, fabric integrity and LLIN survivorship were monitored. Comparisons between nets were tested for significance using the Chi-square test. Exact binomial distribution with 95% confidence intervals (95% CI) was used for percentages. The WHO efficacy criteria used were ≥ 95% knockdown and/or ≥ 80% mortality rate in cone bioassays and ≥ 80% mortality and/or ≥ 90% blood-feeding inhibition in tunnel tests.

**Results:**

At 36 months, Olyset^®^ Plus lost 52% permethrin and 87% PBO content; Olyset^®^ Net lost 24% permethrin. Over 80% of Olyset^®^ Plus and Olyset^®^ Net passed the WHO efficacy criteria for LLINs up to 18 and 12 months, respectively. At month 36, 91.2% Olyset^®^ Plus and 86.4% Olyset^®^ Net survived, while 72% and 63% developed at least one hole. The proportionate Hole Index (pHI) values representing nets in good, serviceable and torn condition were 49.6%, 27.1% and 23.2%, respectively for Olyset^®^ Plus, and 44.9%, 32.8% and 22.2%, respectively for Olyset^®^ Net but were not significantly different.

**Conclusions:**

Olyset^®^ Plus retained efficacy above or close to the WHO efficacy criteria for about 2 years than Olyset^®^ Net (1–1.5 years). Both nets did not meet the 3-year WHO efficacy criteria, and showed little attrition, comparable physical durability and survivorship, with 50% of Olyset^®^ Plus having good and serviceable condition after 3 years. Better community education on appropriate use and upkeep of LLINs is essential to ensure effectiveness of LLIN based malaria interventions.

**Graphical Abstract:**

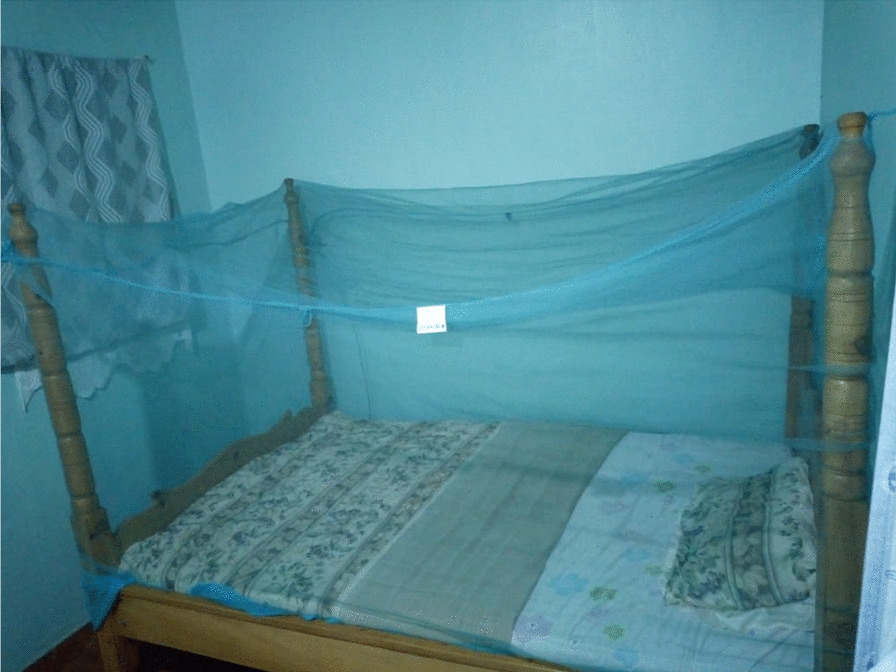

## Background

Insecticide-treated nets reduce child mortality, number of *Plasmodium falciparum* cases and probably the number of *P. vivax* cases per person/year [1]. Factory-produced long-lasting insecticide nets (LLINs) are a core malaria intervention for the global elimination of malaria led by the World Health Organization (WHO) [2]. According to the WHO definition, LLINs should retain efficacy against mosquito vectors for a minimum of 20 standard washes under laboratory conditions and a minimum of 3 years of use under field conditions [3]. When used by most of the people in a population, insecticide-treated nets can protect all people in the community, including those who do not sleep under nets [4]. Currently, the National Malaria Control Programme (NMCP) in Kenya has been scaling up the use of LLINs in malaria endemic and high-risk areas to achieve universal coverage, which is defined by WHO as access to one LLIN for every 1.8 persons in each household [2, 5].

Pyrethroid LLINs were the first brands of treated nets recommended by WHO for malaria control. However, it was believed that insecticide resistance was worsening in Africa and would present a major threat to malaria control [6–8], and that pyrethroid-LLINs were becoming less effective at killing mosquitoes in household conditions when pyrethroid resistance develops [9, 10]. Consequently, piperonyl butoxide (PBO) was incorporated in nets with the intension to improve their efficacy against pyrethroid resistant mosquitoes by acting as a metabolic enzyme inhibitor targeting the P450 cytochrome or mixed function oxidases that metabolise pyrethroids and enhance the efficacy of pyrethroid treated nets. Olyset^®^ Plus has been prequalified by WHO as the first in class PBO net.

However, at the time of beginning the trial due to the absence of guidelines published by WHO of how to evaluate PBO nets, and considering the manufacturer’s product claim, Olyset^®^ Plus was evaluated only as a pyrethroid LLIN against a pyrethroid susceptible mosquito strain rather than against a resistant strain. This paper presents results of a 3-year long-term (Phase III) field trial of the candidate product, Olyset^®^ Plus, along with a positive control product Olyset^®^ Net, in a malaria endemic rice cultivation area of Kirinyaga County, Kenya.

## Methods

The field trial was conducted according to the WHO guidelines [3, 11]. The description of the test products and trial procedures are described below.

### Test products

Bioefficacy and physical durability of the Olyset^®^ Plus and Olyset^®^ Net both manufactured by Sumitomo Chemical were evaluated. The former is a polyethylene mono-filament net incorporated with 2% permethrin corresponding to 20 g permethrin active ingredient (AI)/kg or 800 mg permethrin AI/m^2^ and 1% PBO (corresponding to 10 g PBO AI/kg or 400 mg PBO AI/m^2^). The latter is a polyethylene mono-filament net incorporated with 2% permethrin (w/w) (corresponding to 800 mg permethrin AI/m^2^). Olyset^®^ Net was recommended in 2009 by the WHO Pesticide Evaluation Scheme (WHOPES) for use in malaria control [12], while Olyset^®^ Plus was given interim recommendation by WHO in 2012 with the requirement to evaluate the product in a 3-year long-term trial to confirm its long-lasting efficacy in field [13].

### Trial design and study area

We did a prospective, household randomized controlled, large-scale 3-year field trial in a rice irrigation area of Kirinyaga County, Kenya from 2014 to 2017. The area is located about 100 km northeast of Nairobi at the base of Mt Kenya at an altitude of about 1200 m above sea level [14]. It receives mean annual rainfall of 1200–1600 mm with long rains in March–June and short rains in October–December. Due to high densities of mosquitoes year-round, many local people use mosquito nets. Several bed net trials have previously been conducted here for national product registration. Over 80% of the inhabitants of the area have only primary level education. Most of the houses in the area are made of mud walls with roofs of corrugated iron sheets [14].

Four villages of Maendeleo, Huruma, Kiratina and Kasarani were selected by a simple randomization, of which the first two villages were randomly allocated Olyset^®^ Plus and the other two villages received Olyset^®^ Net (Table 1). In each of the selected village, households were randomly selected.

**Table 1:** Summary of nets distributed in study villages

Village	Population	No. of homesteads	Olyset^®^ Plus		Olyset^®^ Net	
			No. of households	No. of nets given	No. of households	No. of nets given
Kiratina	3190	531	–	–	470	940
Kasarani	1900	380	–	–	300	600
Maendeleo	2810	378	401^a^	802	–	–
Huruma	2425	495	380	760	–	–
Total	10 325	1784	781	1562	770	1540

^a^Some homesteads had more than one household. The households had more than one sleeping area thus multiple nets were distributed in a single household

### Community sensitization and net distribution

Community level meetings were organized with the local leadership (Chiefs, sub-Chiefs and the village heads) and health teams including community health workers, where the objectives of the study were clearly explained. Later, local public meetings (*Barazas*) were organized to sensitize the community about the study. Informed consent to participate in the study was obtained from participating heads of households. A baseline survey and census were conducted in the selected households using a structured questionnaire. Information on size of the family, educational status, occupations, average family income, type of house, number of sleeping places, existing number of nets in each household, their usage pattern, and washing practices were collected. The old nets in use in the households were retrieved and new ones given. Each new net was given a unique identifier and a net master list prepared. Nets were then distributed to the households as per the available sleeping places i.e. one net per sleeping place.

### Net sampling scheme

**Fig. 1:**
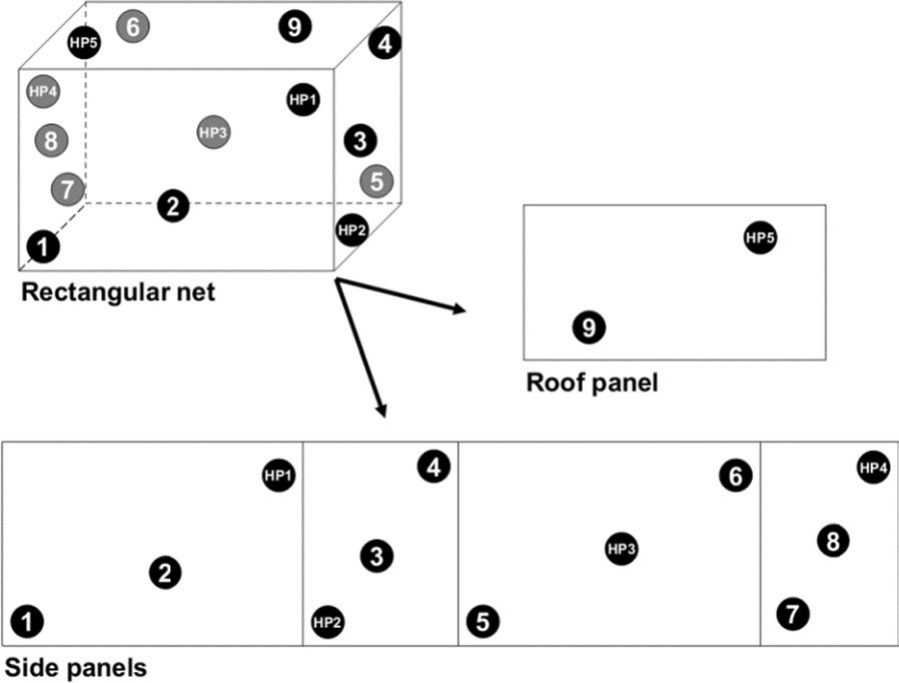
A rectangular net and its individual panels showing positions for cutting netting pieces (positions 1–9 for bioassays; HP1–HP5 for chemical assays)

The scheme of sampling nets at different time points for bioassays and chemical assays is given in Table 2. The netting pieces were cut from the sampled nets from positions 1–9 for bioassays, and positions HP1–HP5 (at time 0) or positions HP2–HP5 (at time 12, 24 and 36 months) for chemical content assays (Fig. 1). All the nets which were sampled and retrieved at the given survey points were replaced with new ones.

**Table 2:** Number of Olyset^®^ Plus and Olyset^®^ Net sampled for assays at different time points during the study

Month	Bioassays and chemical assays	Bioassays alone
0	30	–
6	–	30
12	30	–
18	–	30
24	30	–
30	–	30
36	50	–

### Bioassays

The insecticidal effect of the nets was evaluated using cone bioassays and where required tunnel tests, as per the WHO guidelines [3]. The bioassays were done at time zero (baseline) and then at every 6 months up to 36 months. On each of the netting pieces cut for bioassays, standard WHO plastic cones were held in place using a plastic manifold and a total of 50 laboratory bred, susceptible Kisumu strain *Anopheles gambiae* (non-blood fed, 2–5 day old) were exposed for 3 min (5 pieces per net × 5 mosquitoes per test × 2 replicates). After the exposure, the mosquitoes were removed gently from the cones and kept separately in plastic cups provided with cotton-wool moistened with 10% glucose solution. Knockdown (KD) was recorded at 60 min and mortality at 24 h after exposure. Mosquitoes exposed to untreated polyester net pieces were used as untreated controls. The bioassays were carried out at 27 ± 2 °C temperature and 80% ± 10% relative humidity. When the mosquito knockdown rate was < 95% and mortality < 80%, the mortality and blood-feeding inhibition effect of such nets was assessed in a tunnel test according to the WHO guidelines [3].

For each net, only one netting piece with which mosquito mortality was found closest to the average mortality in the cone test was tested in the tunnel test [3]. One hundred, non-blood fed female *An. gambiae* Kisumu mosquitoes, aged 5–8 days were released at the longer end of a tunnel equipment made of glass. At each end of the tunnel, a 25 cm square cage covered with a polyester netting was fitted. At the shorter end of the tunnel, a rabbit which could not move but was available for mosquito biting was placed. The mosquitoes were released at 18:00 and mosquitoes recovered at 09:00 on the following morning. During the test, the tunnel was kept in a place maintained at 27 ± 2 °C and 80% ± 10% relative humidity in full darkness. Another tunnel with untreated netting was used as control. The mean mortality rate and percentage blood-feeding inhibition were recorded, as follows:

i. Mortality = this was measured by pooling the mortality rates of mosquitoes from the two sections of the tunnel. If mortalities in the control recorded more than 10%, the test was considered invalid.

$${\rm{Blood}} - {\rm{feeding\,inhibition }}\left( \% \right) \, = \, [{1}00 \times \left( {{\rm{Bfc }}-{\rm{ Bft}}} \right)] \, /{\rm{ Bfc}}$$

where, Bfc is the proportion of blood-fed mosquitoes in the control tunnel and Bft is the proportion of blood-fed mosquitoes in the tunnel with the treated net. The treated net was considered to have met the WHO efficacy criteria when mortality in mosquitoes was ≥ 80% and/or blood-feeding inhibition was ≥ 90%.

### Chemical content analysis

The chemical contents in the nets were determined at the beginning and at years 1, 2 and 3 by samplings nets according to the scheme described earlier. The netting pieces cut for determination of active ingredient content were individually rolled up and placed in new, clean and labeled aluminum foils and sent to the Phyto-pharmacy Department of the Walloon Agricultural Research Centre, Gembloux, Belgium, a WHO Collaborating Centre, for chemical analysis.

### Assessment of durability of cohort nets

To monitor durability of nets (i.e. survivorship and changes in physical integrity) during their routine use, 250 each of Olyset^®^ Plus and Olyset^®^ Net were distributed in separate households (2 nets per household) in the same villages by simple randomization. From these cohorts of nets, all nets available at the households at the time of surveys at 6, 12, 24 and 36 months were inspected for their presence and physical integrity. For this, the nets were retracted from the sleeping places, individually hung up on a rectangular frame held outside the house and inspected for presence of any holes on the side and roof panels. The holes were counted for each net, their diameter was measured, and they were classified in the following categories according to the WHO criteria for hole sizes [3, 11]:

Size A1: 0.5–2 cm diameter, Size A2: 2–10 cm diameter, Size A3: 10–25 cm diameter, Size A4: > 25 cm diameter.

The parameters for assessing the integrity of nets included the proportions of Olyset^®^ Plus and Olyset^®^ Net with any size holes or tears, and the proportionate Hole Index (pHI) for each net, which was calculated as follows [11]:

pHI = (1.23 × No. of size A1 holes) + (28.28 × No. of size A2 holes) + (240.56 × No. of size A3 holes) + (706.95 × No. of size A4 holes)

Using the pHI values, Olyset^®^ Plus and Olyset^®^ Net were categorized as those in ‘good condition’ (pHI: 0–64), nets in ‘acceptable’ condition (pHI: 65–642), and torn nets that were likely to provide no protective efficacy (pHI > 642) [15]. The number of nets in ‘good’ and ‘acceptable condition’ together were taken to estimate the survival of nets over time.

The sampled nets were also inspected for any repairs of holes or tears done by the households. After inspection, the nets were returned to the same sleeping place in the same household.

### Household net washing practices

The net washing practices by households were evaluated using a questionnaire at 6, 12, 24 and 36 months after net distribution.

### *Recording of adverse events*

One month after net distribution, a survey was carried out to record adverse events reported by net users, and overall experiences of net usage. A pre-tested questionnaire adapted from the WHO guidelines [11] was administered to the heads or an adult person of the households after obtaining informed consent.

### Statistical analysis

The data collected were entered in excel spreadsheets and cross-checked for accuracy. Data were analyzed using STATA version 14.0 (Stata Corporation, College Station, TX, USA). Comparisons between the two types of nets were tested for significance using the Chi-square (*χ*^2^) test. Exact binomial distribution with 95% confidence intervals (95% CI) was used for percentages. For continuous variables, the arithmetic mean was used depending on the distribution of values compared to a normal distribution.

The nets were considered to have passed the WHO efficacy criteria if the mosquito knockdown rate was found to be ≥ 95% and/or mortality rate ≥ 80% in cone bioassays. For the tunnel tests, nets were considered to have passed the WHO efficacy criteria when mortality in mosquitoes was ≥ 80% and/or blood-feeding inhibition was ≥ 90% [3]. The procedure for calculation of proportionate Hole Index (pHI) for nets is already described above.

### Ethical clearances

Prior to implementing the study, ethical approvals were obtained from the Ethical Research Committee of Kenyatta National Hospital, University of Nairobi (protocol ID P524/8/2013) and the WHO Ethics Review Committee (ID: V2-084). Informed consent of head or an adult person in participating households was obtained in local language. Those who could not read or write were assisted by an independent witness to ensure the study was clearly explained to them and guided them to give a thumb print on the consent form.

## Results

### Demographic characteristics

Among the 1784 participating homesteads with a population of 10 325, a total of 1551 households participated in the study (Table 1). Most of the heads of the households (age: 17–96 years) had received formal education and practiced farming (96.6%).

Out of 100 heads of households with Olyset^®^ Plus interviewed for adverse events, 44% reported itching, 4% eye irritation, and 7% unpleasant smell. Among the Olyset^®^ Net users, 7% each reported nasal discharge and eye irritation. These were reported to be mild symptoms lasting for one or two days. To reduce these effects, 43% users of Olyset^®^ Plus ventilated nets for a day in the open under the sun and 33% under the shade. Among Olyset^®^ Net users, similar actions were taken by 44% and 39%, respectively.

### LLINs wash rate

The findings of the study reported low net wash rates. The reported wash rate was zero at 6 months, 7% each of Olyset^®^ Plus and Olyset^®^ Net washed at least once at 12 months, 12% Olyset^®^ Plus and 11% Olyset^®^ Net washed at least once at 24 months, while 17% Olyset^®^ Plus and 21% Olyset^®^ Net washed at least once at 36 months. In all households where nets had been washed, on 99% occasions, cold water and a local bar soap were used while 1% used washing powder. Of those who washed nets, 86% reported to have dried them under direct sunlight contrary to the advice given to them at the time of distribution to dry them under shade. The results of frequency of washing nets by the users show that about 7–44% nets were washed at least once, and the washing frequency increased in year 2 and 3 (Table 3).

**Table 3:** Frequency of washing nets by the users at different time points

Period (months)	Olyset^®^ Plus				Olyset^®^ Net			
	No. of nets sampled	No. of nets washed once (%)	No. of nets washed twice (%)	No. of nets washed > 2 times (%)	No. of nets sampled	No. of nets washed at once (%)	No. of nets washed twice (%)	No. of nets washed > 2 times (%)
6	30	–	–	–	30	–	–	–
12	30	2 (7)	1 (3)	0	30	2 (7)	0	0
24	30	4 (12)	2 (7)	1 (3)	30	3 (11)	2 (7)	0
36	50	9 (17)	5 (10)	3 (6)	50	11 (21)	8 (6)	3 (6)

The reported wash rate was zero at 6 months

### Bioassay tests

At the baseline, 100% of Olyset^®^ Plus passed the WHO efficacy cut off for LLINs in the cone bioassays i.e., nets causing ≥ 80% mortality and/or ≥ 95% knockdown (KD) in mosquitoes (Table 4). At month 6, 83.3% of Olyset^®^ Plus passed the efficacy cut off by the KD criteria alone. Considering mortality and the blood-feeding inhibition of mosquitoes in tunnel tests for nets failing in cone bioassays, in all 96.7% nets met the WHO efficacy criteria at month 6. Overall, 93.3% and 86.7% (i.e., ≥ 80%) of the sampled Olyset^®^ Plus passed the WHO efficacy criteria at months 12 and 18. Thereafter, the efficacy pass rate declined to 76.7% at month 24 to 42.0% at the end of 36 months of the trial. Thus, Olyset^®^ Plus did not pass the WHO efficacy cut off criteria both at 24 and 36 months of household usage. Olyset^®^ Net performed only up to 12 months; thereafter the pass rates declined below 80%, so the net did not perform as expected at 24 and 36 months. Although each net was classified to have passed or failed the efficacy according to the WHO criteria, no significant difference in the proportions passing efficacy was seen between Olyset^®^ Plus and Olyset^®^ Net [at 6 months (*χ*^2^ = 0.35; *P* = 0.54), 12 months (*χ*^2^ = 1.42; *P* = 0.23), 24 months (*χ*^2^ = 0.72; *P* = 0.39) and 36 months (*χ*^2^ = 0.22; *P* = 0.63)]. The overlapping 95% CI values of the pass percentages for each survey point also show no differences in efficacy of the two nets (Table 4). The data indicate that the entomological efficacy of Olyset^®^ Plus and Olyset^®^ Net in surveys at 24–36 months and 18–36 months, respectively was found to be apparently attributable to their personal protective efficacy, i.e., cumulative effects of KD and mosquito blood-feeding inhibition effects.

**Table 4:** Bioefficacy of Olyset^®^ Plus and Olyset^®^ Net against *Anopheles. gambiae* Kisumu in cone and tunnel tests

Survey month	No. of nets tested	Mean 1 h KD (%)	Mean 24 h mortality (%)	% of nets passed by KD criteria alone^a^	% of nets passed by mortality criteria alone^b^	No. of nets requiring tunnel test	Overall pass rate in percentage (95% CI)^c^
Olyset^®^ Plus							
0	30	99.5	100	100	100	0	100 (80–100)
6	30	97.0	88.2	83.3	73.3	4	96.7 (82–99)
12	30	94.2	74.3	76.7	43.3	6	93.3 (78–99)
18	30	82.9	65.9	36.7	36.7	16	86.7 (69–96)
24	30	77.4	75.3	0.0	46.7	15	76.7 (57–90)
30	30	60.4	56.9	0.0	10.0	27	70.0 (50–85)
36	50	66.4	51.5	2.0	8.0	24	42.0 (28–56)
Olyset^®^ Net							
0	30	99.1	100	100	100	0	100 (88–100)
6	30	96.9	79.8	80.0	60.0	4	93.3 (77–99)
12	30	68.5	54.7	20.0	3.3	24	83.3 (65–94)
18	30	72.1	50.2	30.0	20.0	18	73.3 (54–87)
24	30	79.8	68.9	3.3	46.7	18	66.7 (47–83)
30	30	50.9	50.9	0.0	3.3	29	60.0 (40–77)
36	50	66.3	51.9	0.0	8.0	26	36.0 (23–50)

^a^KD, knockdown ≥ 95%; ^b^mortality ≥ 80%; ^c^taking into account the KD and mortality rates in cone bioassays, and blood-feeding inhibition and mortality rates in tunnel tests

### Chemical contents

At time 0 (baseline), the contents of permethrin and PBO in Olyset^®^ Plus were within the target tolerance limits of 20 ± 5 g/kg and 10 ± 2.5 g/kg, respectively; while the permethrin content in Olyset^®^ Net was also within the target tolerance limit of 20 ± 3 g/kg (Table 5). The within net variation (relative standard deviation, RSD) in permethrin and PBO contents for Olyset^®^ Plus at baseline were 2.2% and 3.7%, respectively, and were within the acceptable tolerance limits. The within net variation in the permethrin content in Olyset^®^ Net at the baseline was 1.2% and was also within the acceptable tolerance limit. At the end of years 1, 2 and 3 of net usage, the permethrin content in Olyset^®^ Plus decreased by 36%, 40% and 52%, and the PBO content showed a marked reduction of 66%, 81% and 87%, respectively. The loss of the permethrin in Olyset^®^ Net was 16% at year 1 and did not change much at year 2 and 3 (19 and 24%, respectively).

**Table 5:** Mean permethrin and PBO contents (relative standard deviation) in nets at baseline, and at 12, 24 and 36 months

Sampling month	Olyset^®^ Plus				Olyset^®^ Net	
	Mean permethrin content in g/kg (95% *CI*)	Permethrin content lost (%)	PBO content in g/kg (95% *CI*)	PBO content lost (%)	Mean permethrin content in g/kg (95% *CI*)	Permethrin content lost (%)
0	18.4 (18.3–18.6)^a^	–	8.98 (8.86–9.10)	–	19.6 (19.5–19.7)^b^	–
12	11.7 (10.6–12.7)	36	3.01 (2.30–3.80)	66	16.5 (16.0–17.0)	16
24	11.1 (10.2–12.0)	40	1.70 (1.20–2.20)	81	15.9 (15.1–16.7)	19
36	8.8 (7.9–9.7)	52	1.16 (0.8–1.5)	87	14.8 (13.9–15.7)	24

^a^Within net variation (relative standard deviation) in permethrin and PBO contents at baseline was 2.2% and 3.7%, respectively (Olyset^®^ Plus). – not applicable, *PBO* piperonyl butoxide

^b^Within net variation (relative standard deviation) in permethrin content at baseline was 1.2% (Olyset^®^ Net)

### Net survivorship and fabric integrity

At the end of year 1, Olyset^®^ Plus reported survivorship rates of 97.2% (*n* = 243), with Olyset^®^ Net recording 99.6% (*n* = 249). At year 2, there were 92.4% (*n* = 231) Olyset^®^ Plus and 90.4% (*n* = 226) of Olyset^®^ Nets still surviving. At the end of the 3 years of study, the survivorship rates for Olyset^®^ Plus and Olyset^®^ Net were at 91.2% (*n* = 228) and 86.4% (*n* = 216), respectively.

The pHI values of the nets showed that 87.6% of Olyset^®^ Plus were in good condition at 6 months, 7.6% were in acceptable/serviceable condition, and 4.8% were too torn (Table 6). These proportions increased gradually during the study and at 36 months, 49.6% were in good condition, 27.1% in acceptable/serviceable condition, and 23.2% were too torn. A similar trend of attrition was seen for Olyset^®^ Net, with 44.9%, 32.8% and 22.2% nets being in good condition, acceptable condition or too torn at 36 months, respectively.

**Table 6:** Progression of hole formation and proportionate Hole Index (pHI) values for Olyset^®^ Plus and Olyset^®^ Net

Net brand	Follow up months	No. of nets sampled	Number of nets with any size holes (%; 95% *CI*)	No. of nets by pHI values (%; 95% *CI*)		
				pHI < 65 (in good condition)	pHI 65–642 (in acceptable condition)	pHI > 642 (too torn nets)
Olyset^®^ Plus	6	250	115 (46.0; 39.9–52.1)	219 (87.6; 82.9–91.1)	19 (7.6; 4.9–11.5)	12 (4.8; 2.8–8.2)
	12	243	131 (54.0; 47.6–60.1)	190 (78.2; 72.6–82.9)	30 (12.4; 8.8–17.0)	23 (9.5; 6.3–13.8)
	24	231	120 (52.0; 45.5–58.3)	145 (62.8; 56.7–68.7)	58 (25.1; 19.9–31.1)	28 (12.0; 8.5–16.9)
	36	228	144 (63.0; 56.7–69.1)	113 (49.6; 43.1–56.0)	62 (27.1; 21.8–33.3)	53 (23.2; 18.2–29.2)
Olyset^®^ Net	6	250	157 (63.0; 56.7–68.6)	186 (74.4; 68.7–79.4)	57 (22.8; 18.1–28.4)	7 (2.8; 1.3–5.7)
	12	249	169 (68.0; 61.8–73.4)	159 (63.9; 57.7–69.6)	76 (30.5; 25.1–36.5)	14 (5.6; 3.4–9.2)
	24	226	147 (65.0; 58.6–70.9)	120 (53.1; 46.6–59.5)	71 (31.4; 25.7–37.7)	35 (15.5; 11.4–20.8)
	36	216	156 (72.0; 65.9–77.9)	97 (44.9; 38.4–51.6)	71 (32.8; 26.9–39.4)	48 (22.2; 17.2–28.2)

Data on the comparison of estimates of the mean pHI values and the mean hole area for Olyset^®^ Plus and Olyset^®^ Net are given in Table 7.

**Table 7:** Comparison of the estimates of the mean pHI and the mean hole area for Olyset^®^ Plus and Olyset^®^ Net

Survey month	Olyset^®^ Plus			Olyset^®^ Net		
	No. of nets inspected	Mean hole area in cm^2^ (± SD)	Mean pHI (± SD)	No. of nets inspected	Mean hole area in cm^2^ (± SD)	Mean pHI (± SD)
6	250	52.3 ± 230.5	93.3 ± 313.6	250	123.3 ± 272.3	110.5 ± 221.8
12	243	229.2 ± 574.2	153.6 ± 437.0	249	192.5 ± 376.2	156.8 ± 306.5
24	231	389.8 ± 975.4	317.6 ± 794.7	226	873.9 ± 353.4	279.4 ± 428.1
36	228	630.7 ± 1191.8	513.9 ± 970.9	216	621.5 ± 1710.3	506.4 ± 1393.2

*pHI* proportionate Hole Index

The proportion of Olyset^®^ Plus nets having been repaired by households increased from 6% (95% CI: 3.4–9.7) at month 6 to 17.9% (95% CI: 13.2–23.6) at months 36, while that of Olyset^®^ Net increased from 5% (95% CI: 2.8–8.7) to 12% (95% CI: 8.0–17.1) in the same period.

## Discussion

The study evaluated the efficacy of Olyset^®^ Plus incorporated with permethrin and in a 36-month long field trial against *An. gambiae* Kisumu, a susceptible strain. Olyset^®^ Net incorporated with permethrin alone was included in the trial as a positive control net. A net was considered to have passed the WHO efficacy criteria if it caused ≥ 80% mortality and/or ≥ 95% knockdown in cone bioassays or ≥ 80% mortality and/or ≥ 90% blood-feeding inhibition in tunnel tests [3]. Most of the Olyset^®^ Plus and Olyset^®^ Net lost the knockdown effect during the 24–36 months follow-up. Overall, neither Olyset^®^ Plus nor Olyset^®^ Net performed as they should even after 2 years and did not meet the WHO efficacy criteria of a 3-year LLIN.

Previous studies have demonstrated enhanced efficacy of Olyset^®^ Plus against resistant *An. gambiae* attributable to PBO [16–18]. A recent randomised controlled field trial in Tanzania with high levels of pyrethroid resistance in malaria vectors *An. gambiae* and *An. arabiensis* also attributed higher impact on malaria of Olyset^®^ Plus over Olyset^®^ Net to the presence of PBO that was sustained after 21 months of the trial [19]. However, there is a view that these two nets are not comparable and that the higher efficacy of Olyset^®^ Plus was due to much more bleed rate of permethrin into its surface and not due to PBO that was incorporated in small amount and dwindled rapidly over time [20]. In this trial, Olyset^®^ Plus was evaluated as a pyrethroid LLIN since there were no guidelines published by WHO of how to evaluate PBO nets at the time of this trial, and considering the manufacturer’s product claim.

While, it was reported that high intensity insecticide resistance will reduce the level of personal and community protection of pyrethroid LLINs [21] and PBO nets could be effective against malaria in some resistance scenarios [22], a recent WHO multi-country study found no evidence of an association between insecticide resistance and malaria infection prevalence or incidence [23]. There is also a view that at present there is limited evidence that recent reduction in the impact on malaria in sub-Saharan Africa was due to increasing resistance in malaria vectors to the pyrethroid insecticides used in treating nets [24].

The initial permethrin content in both nets was more or less similar but the enhanced efficacy of Olyset^®^ Plus was mainly due to the higher release of permethrin into the surface fibres and presence of PBO than Olyset^®^ Net. The gradual decline in efficacy of both nets is consistent with the loss of permethrin and PBO in Olyset^®^ Plus and of permethrin in Olyset^®^ Net over time during their routine usage by the community. The decline in permethrin content was more rapid in Olyset^®^ Plus than Olyset^®^ Net. This explains why Olyset^®^ Plus having permethrin and PBO was relatively superior than Olyset^®^ Net initially, i.e., until 18 months, but after that their performance was below the WHO efficacy cut off. While the PBO content in Olyset^®^ Plus at the baseline was within the target tolerance limits, 66% of the PBO content was lost within one year of net usage, and the loss increased to 81% at year 2 and 87% at year 3. Previous studies have reported that the insecticidal content in LLINs decay over time [6]. While permethrin and PBO contents may be lost due to natural decay and evaporation, washing of nets frequently and drying them under direct sunlight are known to contribute to such losses [25, 26]. In this study, despite manufacturer’s label recommendation not to expose nets to direct sunlight, a good proportion of both type of nets were exposed to bright sunlight before use and left to dry in the sun after washing. This would have reduced both permethrin and PBO contents and consequently reduced the biological efficacy of these nets and should be borne in mind when considering these results. Even though Kisumu strain was tested, the loss of PBO would have affected efficacy of Olyset Plus as PBO can improve net performance even against susceptible strains, which too have P450s, and PBO is also known to act as a good solvent that can aid cuticular penetration and help improve insecticidal activity. The operational implication of rapid loss of PBO is that Olyset^®^ Plus was unlikely to remain effective even against pyrethroid resistant mosquitoes for 2 or 3 years of use.

Community net wash practices were comparable between the two nets. During the first 6 months, no net was reported to have been washed. At 1 year, 7% of Olyset^®^ Plus and 7% of Olyset^®^ Net were reported to have been washed at least once. At month 36 months, about 17% and 21% of Olyset^®^ Plus and Olyset^®^ Net respectfully had been washed at least once. LLINs are usually recommended to be washed at most every 3 months [5], although this frequency depends on local cultural practices and water availability. Different net washing frequencies have been reported in other studies spanning from a high of 8 washes per month in Mali [27] to 4–7 times in Tanzania [28] to a low average of 1.5 washes per year in Uganda [29]. In coastal Kenya, on average nets were washed twice in 6 months, with blue colour nets least often washed than white or green colour nets, and older nets washed more frequently [30]. In western Kenya lowlands, three quarter of nets were washed once within 3 months and a third of them were dried under the sun [31]. The main reasons for low reported wash rates during the annual surveys in our study could be attributed to net users’ poor recall of the net washing frequencies, and people’s apprehension that washing of nets reduce net efficacy, although there was no water scarcity in the trial area being located in a rice irrigation area.

The study participants reported experiencing certain transient adverse effects during the first two days of use of nets, but these could be prompted reactions since the community members had been informed about the possibility of such effects at the time of net distribution. The higher side effects from use of Olyset^®^ Plus is in line with the higher release of permethrin as mentioned above. Users resorted to ventilating their nets in direct sunlight to reduce the effects although our project team had advised to dry up and keep the nets under shade. Ventilating new nets or drying washed nets under direct sunlight might have contributed to a significant reduction in active ingredient content and bio-efficacy of nets.

At the end of the 3-year period, among the two cohorts of 250 nets each, only about 9% Olyset^®^ Plus and about 14% Olyset^®^ Net were reported to have been lost showing a high net survivorship. These results are in line with the expected 75% net survivorship rate reported by the NetCALC 3-year serviceable prediction model [32]. Previous studies have reported varying rate of net survivorship in African countries, such as 58% nets were lost within two years in Rwanda [33], only 39% of distributed nets remained both present and in serviceable physical condition 2–4 years after a mass campaign in Tanzania [34], but in Western Uganda an estimated attrition rate of just 12% after three years of use was observed for a polyester net [35]. In four African countries of Mozambique, Nigeria, DRC and Zanzibar, Tanzania, survival in serviceable condition of polyethylene and polyester LLINs after 31–37 months of use varied between different sites from 17 to 80% with median survival from 1.6 to 5.3 years [36]. In our study, the observed low attrition rate of nets appears to be also due to the ‘Hawthorne effect’ as the households included in the two cohorts of nets were visited every 6 or 12 months to inspect the nets. So, the awareness among study participants that the nets were regularly being followed could have modified their behaviour in favour of more careful upkeep and maintenance of the nets and as such may have been a limitation in this study.

Studies have shown that despite small to medium size holes (64 < PHI < 642), LLINs are still protective against mosquito bites due to the excito-repellent effects of insecticides [35]. However, the nets may become ineffective when the overall hole area in nets reaches a certain threshold [37]. In our study, both Olyset^®^ Plus and Olyset^®^ Net recorded high fabric integrity up to the 3-year trial period. At 12 months after distribution, 78.1% of Olyset Plus and 63.8% of Olyset^®^ Net were in good/acceptable condition, 12.3% Olyset^®^ Plus and 30.5% Olyset^®^ Net were in serviceable condition, while 9.4% Olyset^®^ Plus and 5.6% Olyset^®^ Net were completely torn and required to be replaced. At the end of the 3-year trial period, 49.5% of Olyset^®^ Plus and 44.9% of Olyset^®^ Net were in good/acceptable condition, 27.1% Olyset^®^ Plus and 32.8% Olyset^®^ Net in serviceable condition, while 23.2% Olyset^®^ Plus and 22.2% Olyset^®^ Net were completely torn and required replacement.

Although Olyset^®^ Net retained a higher permethrin content that Olyset^®^ Plus, the latter showed longer duration of efficacy than the former, i.e., 18 months compared with 12 months above the WHO efficacy cut off, and higher efficacy thereafter i.e., the efficacy of Olyset^®^ Plus declined to 42% at the end of 3-year trial period and that of Olyset^®^ Net to 36% over the same period. This could be attributed to higher release of permethrin into the netting surface, and also because PBO can make nets perform better even against susceptible mosquitoes. Pyrethroid-PBO nets have previously been associated with higher mosquito mortality and lower blood-feeding rates in areas of high-level insecticide resistance than were non-PBO LLINs [38, 39]. Although in our study we did not test nets with a pyrethroid resistant mosquito strain, it is likely that the fast decline of PBO in Olyset^®^ Plus was caused by the inappropriate exposure to sunlight, as well as the normal loss through washing would have reduced efficacy against resistant mosquitoes in the same way that it reduced efficacy against the susceptible mosquitoes tested here.

Appropriate testing guidelines and field studies are thus required to evaluate the operational effectiveness of PBO nets against pyrethroid resistant mosquitoes and considering the PBO retention and release properties. The revised WHO LLIN guidelines should clarify for how long PBO nets should be effective and if the current WHO efficacy criteria for a 3-year LLIN should be reduced to fit the chemistry.

A limitation of our trial was that we did not evaluate efficacy of Olyset^®^ Plus against resistant *An. gambiae* mosquitoes. Instead, we tested its efficacy for the presence of permethrin alone using a susceptible malaria vector strain, although inclusion of PBO in nets has the potential of increasing insecticidal efficacy due to increased cuticular penetration and presence of P450s in susceptible insects. There is thus a need for suitable testing guidelines and a much more validation of the impact of PBO in pyrethroid-PBO nets.

## Conclusions

Olyset^®^ Plus did not meet the WHO criteria for efficacy of LLINs in this 3-year Phase III study, which could have resulted from faster loss of permethrin and PBO and users not following the manufacturer’s recommendations to not leave the nets under direct sunlight. Better community education is therefore essential to ensure appropriate use and upkeep of nets in areas with LLIN-based interventions in order to maximize their operational impact on the prevention, control and elimination of malaria. Failure to do this would result in operational implications requiring early replenishment with new nets to ensure that such an LLIN based malaria intervention remains effective.

## Data Availability

All the relevant datasets supporting the conclusions of this article are included within the article.

## Abbreviations

*CI*: Confidence interval
CHW: Community Health Workers
KD: Knockdown
LLIN: Long-lasting insecticidal nets
NMCP: National Malaria Control Programme
pHI: proportionate Hole Index
PBO: Piperonyl butoxide
RSD: Relative standard deviation
SD: Standard deviation
WHO: World Health Organization
